# Novel spiral mapping catheter facilitates observation of the time-to-pulmonary vein isolation during cryoballoon ablation

**DOI:** 10.1007/s00380-018-1254-x

**Published:** 2018-10-09

**Authors:** Alexander Pott, Kerstin Petscher, Michael Baumhardt, Tilman Stephan, Manuel Rattka, Rima Paliskyte, Carlo Bothner, Mirjam Keßler, Wolfgang Rottbauer, Tillman Dahme

**Affiliations:** Department of Medicine II, Ulm University Medical Center, Albert-Einstein-Allee 23, 89081 Ulm, Germany

**Keywords:** Atrial fibrillation, Cryoballoon, Spiral mapping catheter, Time-to-isolation, Achieve advance

## Abstract

Observation of the time-to-pulmonary vein isolation (TTI) by a spiral mapping catheter has emerged as a valuable procedural parameter in cryoballoon pulmonary vein isolation (PVI). The 1st generation spiral mapping catheter (Achieve, SMC1) has been available as an 8-polar catheter with a distal loop diameter of 15 or 20 mm. The novel spiral mapping catheter (Achieve Advance, SMC2) was designed as a true guidewire and is available, in addition to the sizes of the SMC1, as a 10-polar mapping catheter with a distal loop diameter of 25 mm. Whether these novel features of SMC2 influence procedural characteristics of Cryo-PVI in comparison to SMC1 has not been reported. In this prospective cohort study 158 patients (age 65.1 ± 12.4 years, female 39%, paroxysmal AF 60%) undergoing PVI with the 2nd generation cryoballoon were included. SMC1 was used in 57 patients (36%), whereas 101 patients (64%) underwent Cryo-PVI with the SMC2. All PVs (623/623, 100%) were isolated successfully. Mean procedure duration was 72.0 ± 18.9 min in the SMC1 group and 74.4 ± 19.1 min in the SMC2 group (*p* = 0.432). Mean fluoroscopy time was also not different between both study groups (SMC1 15.7 ± 6.6 min, SMC2 15.7 ± 7.3 min, *p* = 0.593). TTI was observed in 68.6% of pulmonary veins in the SMC1 group, whereas TTI observation rate was 82.6% in the SMC2 group (*p* < 0.001). Number of freezes (5.5 ± 1.5 vs. 6.5 ± 1.9; *p* = 0.001) and total freeze duration (14.1 ± 4.5 vs. 17.6 ± 5.6; *p* < 0.001) were increased in the SMC2 group. SMC2 significantly increases TTI observation rate during Cryo-PVI. Procedure duration and fluoroscopy time are similar and number of freezes and total freeze duration are increased compared to PVI with SMC1 due to decreased stability and maneuverability of SMC2.

**Electronic supplementary material:**

The online version of this article (10.1007/s00380-018-1254-x) contains supplementary material, which is available to authorized users.

## Introduction

**Fig. 1:**
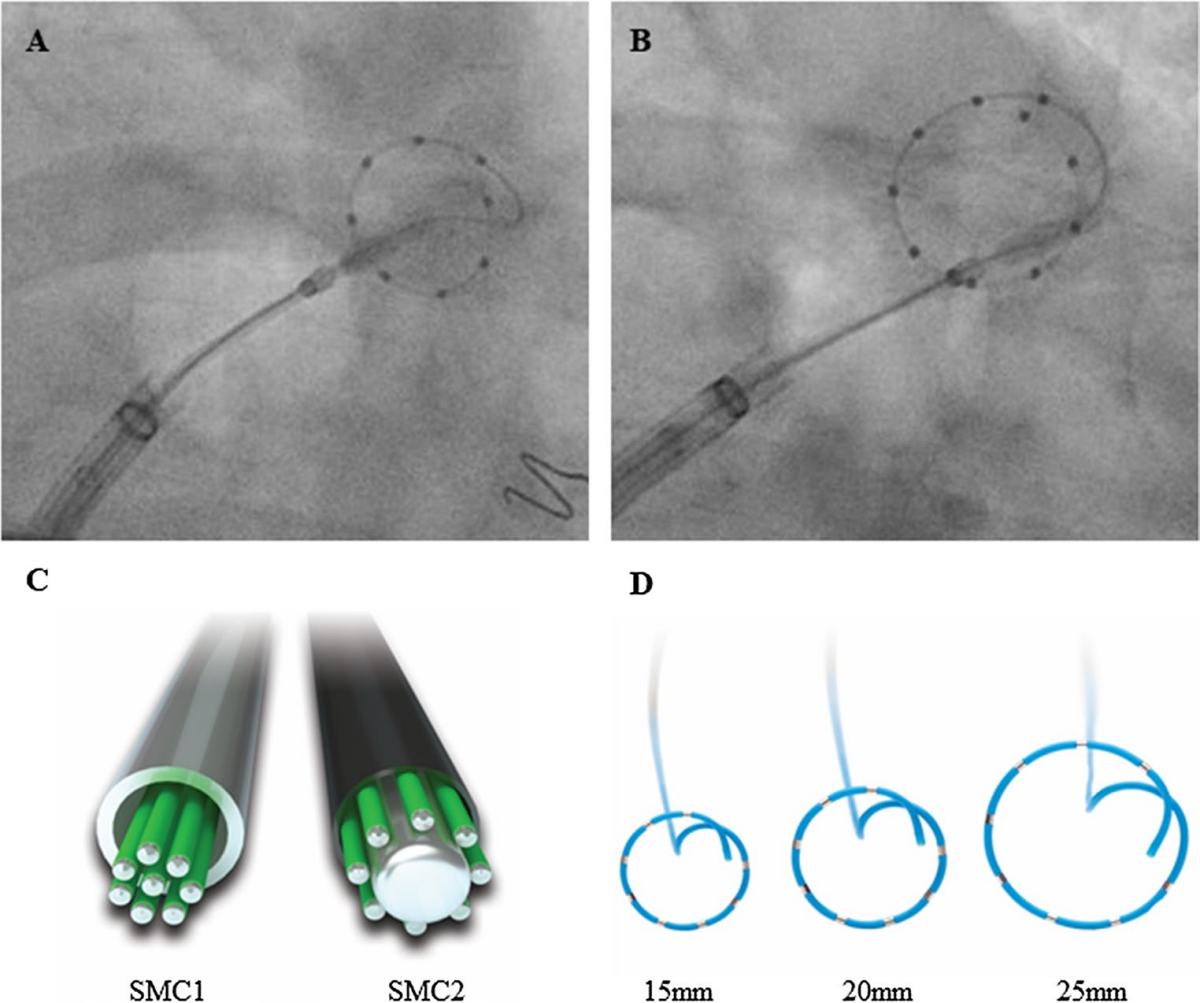
Inflated arctic front advance cryoballoon with spiral mapping catheter introduced through the balloon inner lumen and positioned in the PV at the closest achievable proximity to enable real-time PV potential observation. **a** 1st generation 8-polar spiral mapping catheter (Achieve, SMC1) with 20 mm diameter. **b** 2nd generation 10-polar spiral mapping catheter (Achieve Advance, SMC2) with 25 mm diameter and 10 electrodes enables more proximal electrode localization at the PV ostium. Fluoroscopic images recorded in RAO 30°. **c** The SMC1 was constructed with a steel jacket containing electrical wires. SMC2 was designed without a steel jacket, instead SMC2 was designed with a solid core wire and electrical wires are put around in a polyimite coating. **d** SMC1 and SMC2 are available as 15 mm and 20 mm diameter and 8 electrodes. SMC2 is available in addition as 25 mm diameter and with 10 electrodes

Pulmonary vein isolation (PVI) is the cornerstone in the treatment of patients with symptomatic atrial fibrillation [1–4]. During PVI, recording and evaluation of pulmonary vein (PV) potentials significantly contributes to an effective ablation procedure [5]. Real-time observation of pulmonary vein isolation with a spiral mapping catheter (Achieve, Medtronic Inc, Minneapolis, MN, SMC1, Fig. 1a) placed in the pulmonary vein via the inner lumen of the cryoballoon catheter has emerged as a valuable procedural parameter to predict freedom from atrial fibrillation during cryoballoon PVI. A short time-to-isolation (TTI) is a predictor of low recurrence rates, whereas a long TTI is linked to high PV reconnection rates [6–8]. Furthermore, a high rate of TTI observation enables individual titration of cryoenergy and thereby reduces procedure duration and fluoroscopy time [9–13].

SMC1 has been available as an 8-polar catheter with a distal loop diameter of 15 or 20 mm. The 2nd generation of the spiral mapping catheter (Achieve Advance, Medtronic Inc, Minneapolis, MN, SMC2), has been designed as a true guidewire by abandoning the steel jacket of SMC1 which contained the electrical wires and instead putting the electrical wires around a core wire. This design changes lead to a much more flexible shaft. SMC2 is available, in addition to the sizes of the SMC1, as a 10-polar mapping catheter with a distal loop diameter of 25 mm (Fig. 1b–d). To date, no data on efficacy of PVI with SMC2 compared to SMC1 have been reported. This is to the best of our knowledge the first study evaluating the impact of SMC2 on cryoballoon PVI in comparison to SMC1.

## Methods

### Study population

In this prospective cohort study, we compared the first 41 patients, who underwent PVI for the treatment of paroxysmal or persistent atrial fibrillation with the 2nd generation cryoballoon (Arctic Front Advance, Medtronic Inc, Minneapolis, MN, USA) in combination with the SMC2 to the last 101 patients treated with the 2nd generation cryoballoon and SMC1. Exclusion criteria were long-standing persistent AF, previous left atrial (LA) ablation, LA diameter > 55 mm, uncontrolled heart failure (NYHA class IV) and severe valvular disease. The study complies with the Declaration of Helsinki and was approved by our institutional review committee. All patients gave written informed consent to the procedure and to the participation in this observational study.

### Aim of the study

We sought to evaluate the impact of SMC2 on procedure duration, fluoroscopy time, TTI observation rate and complication rate in patients during cryoballoon PVI in comparison to SMC1.

### Periprocedural management

Intracardiac thrombi were ruled out in every patient by transesophageal echocardiography prior to PVI. Additional preprocedural imaging was not applied. Patients on vitamin K antagonists were scheduled for the procedure at a target INR of 2.0–3.0, whereas patients on non-vitamin K antagonist oral anticoagulants (NOACs) were advised to hold medication 24 h prior to the procedure. NOACs were continued on the evening of the day of the procedure. Anticoagulation was continued for at least 2 months following the procedure in all patients. Pericardial effusion was routinely ruled out by echocardiography immediately after the procedure and prior to hospital discharge.

### Cryoballoon ablation procedure

Cryoballoon ablation with SMC1 has been described in detail before [9, 14]. Briefly, the cryoballoon was introduced to the left atrium via a steerable transseptal sheath (Flexcath Advance, Medtronic, Minneapolis, MN, USA) after single transseptal puncture. In the SMC2 group the selection of the diameter of the spiral mapping catheter (20 or 25 mm) was at the operator’s discretion in the first 41 cases based on PV diameter estimated by PV angiography. In the last 60 cases in the SMC2 group the 25 mm spiral mapping catheter was used exclusively. After balloon inflation and placement at the PV ostia, the spiral mapping catheter was positioned in the PV at the closest achievable proximity to the ostium to enable real-time observation of PV potentials during PV isolation. PV occlusion was documented by injection of contrast medium. The TTI was defined as the time of the last recording of a PV potential before sustained isolation. In both study groups individualized TTI-dependent titration of cryoenergy was performed. In case of a TTI < 30 s the total freeze cycle duration was decreased to 120 s and no bonus freeze was applied. If TTI was between 30 and 60 s a single 180 s freeze cycle was applied. If TTI was > 60 s a 180 s freeze cycle followed by a 180 s bonus freeze was applied. Also, if no TTI could be documented, but isolation was achieved, a single 180 s freeze cycle was applied followed by a 180 s bonus application of cryoenergy. Isolation of all PVs was reassessed at the end of the procedure by documentation of entrance- and exit-block except at PVs with a TTI ≤ 60 s. Cryoenergy application was aborted if luminal esophageal temperature (LET) measured by transnasally inserted temperature probe (S-CATH; Circa Scientific Inc., Englewood, CO, USA) decreased below 20 °C [15].

### Statistical analysis

Significance of differences of numeric values between the two groups was calculated by *t* test if normal distribution with equal variance was given. Normal distribution was determined by Shapiro–Wilk test and equal variance by Brown–Forsythe test. Numeric variables that were not normally distributed were analyzed by Mann–Whitney rank sum test. Categorical variables were analyzed by Chi-square test or Fisher’s exact test. A *p* value < 0.05 was considered significant. Statistical assessment was performed with Excel (Version 2016, Microsoft Inc., Redmond, WA, USA) or XLStat software (V 2016.02.28430, Addinsoft, New York, NY, USA).

## Results

### Study population

We included 158 patients (mean age 65.1 ± 12.4 years, female 39%, paroxysmal AF 60%, mean CHA_2_DS_2_-VASc-Score 2.8 ± 1.6) undergoing cryoballoon PVI with the second generation cryoballoon. In 57 patients (36%) the SMC1 was applied (SMC1 group) and 101 patients (64%) underwent cryoballoon PVI with the SMC2 (SMC2 group). Baseline characteristics did not differ significantly between both study groups (Table 1). SMC1 was exclusively used with a diameter of 20 mm, SMC2 with a 20 mm diameter was applied in 12 patients, SMC2 with a 25 mm diameter was used in 89 patients (Table 2).

**Table 1:** Patient baseline characteristics

	SMC1	SMC2	*p* value
Patients (*n*)	57	101	
Gender, female [*n* (%)]	25 (43.8)	37 (36.6)	0.469
Age (years)	64.1 ± 11.3	65.6 ± 13.0	0.303
Paroxysmal Afib [*n* (%)]	35 (61.4)	60 (59.4)	0.939
CHA_2_DS_2_-VASc-Score	2.8 ± 1.5	2.8 ± 1.7	0.714
Heart failure [*n* (%)]	21 (36.8)	37 (36.6)	0.884
Arterial hypertension [*n* (%)]	42 (73.7)	70 (69.3)	0.690
Diabetes mellitus [*n* (%)]	8 (14.0)	12 (11.9)	0.804
LA diameter (mm)	45 ± 7	44 ± 8	0.552

**Table 2:** Procedural characteristics

	SMC1	SMC2	*p* value
Mapping catheter size [*n* (%)]			
20 mm (8 electrodes)	57 (100)	12 (12)	
25 mm (10 electrodes)	0 (0)	89 (88)	
Procedure duration (min)	72.0 ± 18.9	74.4 ± 19.1	0.432
Fluoroscopy time (min)	15.7 ± 6.6	15.7 ± 7.0	0.593
PVs isolated [*n* (%)]	226 (100)	397 (100)	1.000
Freezes per patient (*n*)	5.5 ± 1.5	6.5 ± 1.9	0.001
Freeze duration per patient (min)	14.1 ± 4.5	17.6 ± 5.6	< 0.001
Freeze abortion [*n* (%)]	4 (1.3)	32 (4.9)	0.010
PNP	4	7	0.981
LET	1	11	0.117
Balloon dislodgment	0	4	0.314
Ineffectiveness	0	9	0.036
Mean TTI all PVs (s)	44.2 ± 24.6	45.9 ± 27.4	0.834
TTI LSPV	49.9 ± 25.8	48.4 ± 20.6	0.532
TTI LIPV	30.5 ± 13.5	36.9 ± 18.4	0.130
TTI RSPV	40.6 ± 18.1	38.1 ± 17.6	0.627
TTI RIPV	53.8 ± 31.4	56.1 ± 40.5	0.637
Complications [*n* (%)]			
Pericardial tamponade [*n* (%)]	0 (0)	1 (1)	1.000
Groin hematoma [*n* (%)]	1 (1.8)	0 (0)	0.361
Other [*n* (%)]	0 (0)	0 (0)	1.000

### Procedural results

**Fig. 2:**
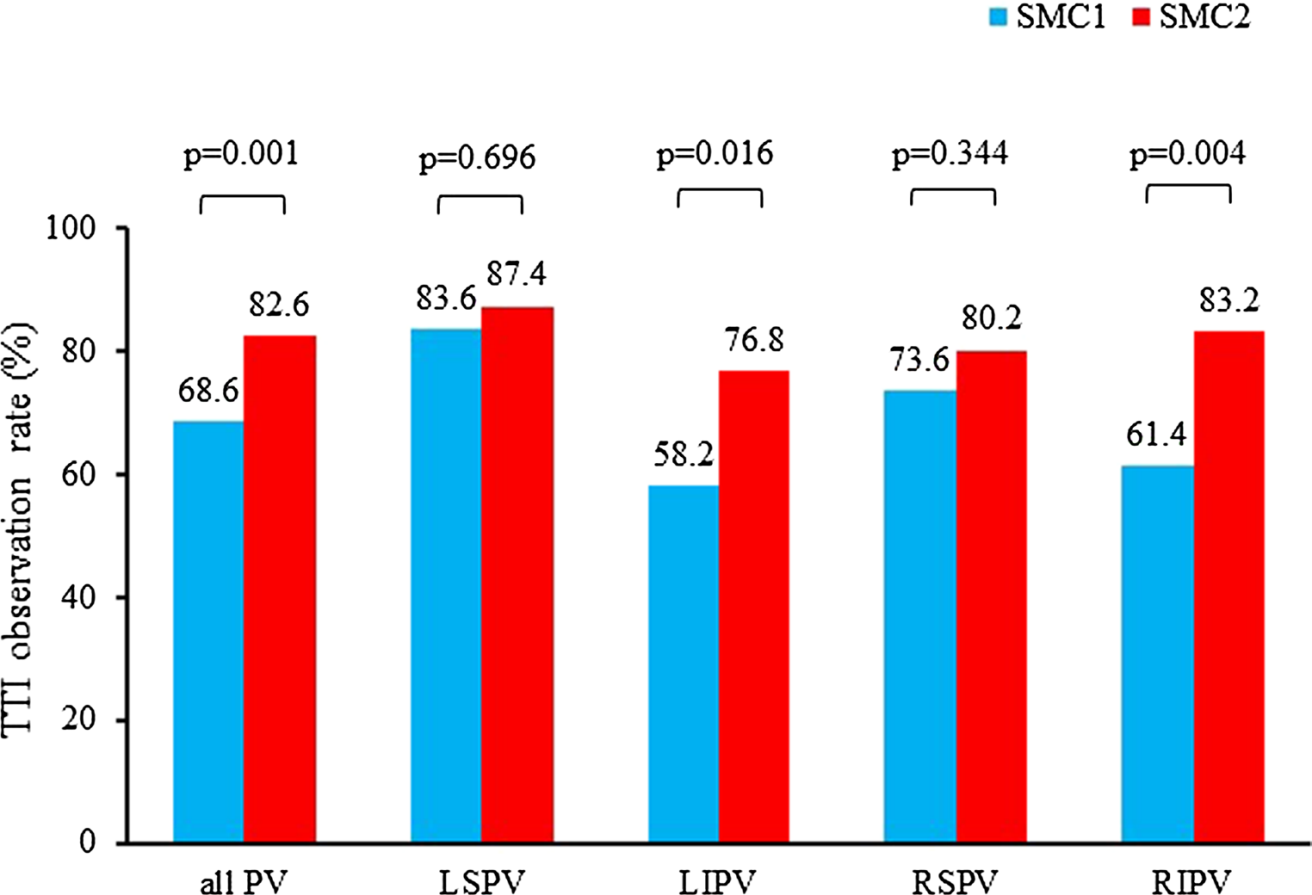
Overall TTI observation rate and per vein depending on spiral mapping catheter. Using the SMC2 leads to significantly higher overall TTI observation rate and in the LIPV and RIPV, whereas TTI observation rate of the upper PV was comparable in the SMC1 and SMC2 group

In the SMC1 group we identified 226 PVs and 397 PVs were identified in the SMC2 group. All PVs (623/623, 100%) were isolated successfully with the SMC1 or SMC2 placed in the lumen of the 2nd generation cryoballoon. Exchange of the mapping catheter to a stiff guidewire was necessary in one case in the SMC2 group. Overall TTI observation rate in the SMC1 group was 68.6% (155/226 PVs), whereas TTI observation rate for all PVs was 82.6% (328/397 PVs) in the SMC2 group (*p* = 0.001). We were able to determine the time-to-isolation in 46/55 LSPVs (83.6%) in the SMC1 group and in 83/94 LSPVs (88.3%) in the SMC2 group (*p* = 0.696), in 32/55 LIPVs (58.2%) in the SMC1 group versus 73/94 LIPVs (77.7%) in the SMC2 group (*p* = 0.002), in 42/57 RSPVs (73.6%) in the SMC1 group compared to 81/101 RSPVs (80.2%) in the SMC2 group (*p* = 0.344) and in 35/57 RIPVs (61.4%) in the SMC1 group versus 84/101 RIPVs (83.2%) in the SMC2 group (*p* = 0.004, Fig. 2). No TTI was registered in 2 LCPVs in the SMC1 group, while in 3 out of 7 LCPVs a TTI was registered in the SMC2 group (*p* = 0.500).

We further analyzed the TTI observation rates with regard to the diameter of spiral mapping catheter in the 69 patients treated with a 20 mm catheter (SMC1 and SMC2) versus the 89 patients treated with a 25 mm catheter (only SMC2). While TTI observation rate with the 20 mm catheter was 70.2% (191/272 PVs), TTI was significantly more often registered with the 25 mm diagnostic catheter (83.2%, 292/351 PVs, *p* < 0.001), indicating that catheter size and additional electrodes facilitate registration of PV potentials during PV isolation. Accordingly, TTI observation rate between SMC1 (68.6%) and 20 mm SMC2 (78.2%) was statistically not different (*p* = 0.258). Comparison of TTI observation rate between SMC2 with 20 mm and 25 mm demonstrated a numerically higher real-time observation rate using a larger mapping catheter (20 mm: 78.2% vs 25 mm: 83.2%). However, this difference is statistically not significant (*p* = 0.407).

In contrast to the increased rate of observed real-time PV isolations with the SMC2 the actual time-to-isolation (TTI) did not differ between both groups. Mean time-to-isolation was 44.2 ± 24.6 s in the SMC1 group and 45.9 ± 27.4 s in the SMC2 group (*p* = 0.834). In accordance, there was also no significant difference in the mean TTI of each PV between both study groups.

However, incidence of prematurely aborted freezes caused by phrenic nerve palsy (PNP), low oesophageal temperature (LET) < 20 °C, cryoballoon dislodgment or ineffective pulmonary vein isolation was significantly higher when the 2nd generation spiral mapping catheter was used (SMC1: 4/314 freezes (1.3%) vs. SMC2: 32/655 freezes (4.9%; *p* = 0.009). Especially, ineffective pulmonary vein isolation leading to premature freeze abortion was observed more frequently in the SMC2 group compared to the control group [SMC1: 0/314 freezes (0%) vs. SMC2: 9/655 freezes (1.4%; *p* = 0.036)]. Similarly, freeze abortion rate caused by LET < 20 °C tended to be higher when SMC2 was used although this was statistically not different (*p* = 0.120, Table 2).

Remarkably, mean total freeze duration per patient was significantly longer in the SMC2 group (17.6 ± 5.6 min) compared to the SMC1 group (14.1 ± 4.5 min, *p* < 0.001). Similarly, mean number of freezes per patient was significantly higher in the SMC2 study group with 6.5 ± 1.9 compared to 5.5 ± 1.5 in the SMC1 group (*p* = 0.001).

The shaft of the SMC2 is much softer compared to SMC1, which leads to decreased maneuverability in patients with difficult PV anatomies. Specifically, the softer shaft precludes placement of the spiral tip containing the electrodes of SMC2 inside the PV in certain difficult anatomies, especially tortuous PVs (supplementary online movie 1 and 2). In these cases, backup can be increased by advancing the cryoballoon catheter over the soft shaft of the SMC2 catheter into the PV (supplementary online movie 3 and 4). Nevertheless, decreased maneuverability of SMC2 did not translate into significantly increased procedure duration or fluoroscopy time. Mean procedure duration was 72.0 ± 18.9 min in the SMC1 group and 74.4 ± 19.1 min in the SMC2 group (*p* = 0.432). Mean fluoroscopy time was 15.0 ± 5.7 min in the SMC1 group and 15.7 ± 7.3 min in the SMC2 group (*p* = 0.593). Procedural details and acute ablation results are shown in Table 2.

### Complications

Transient phrenic nerve palsy occurred in 11 patients of the whole study group with 4 PNP (5.8%) in the SMC1 group and 7 PNP (6.9%) in the SMC2 group (*p* = 1.0). All PNP resolved completely by the end of the procedure. No periprocedural stroke or systemic embolism occurred. Groin hematoma (without blood transfusion) was observed in one patient in the SMC1 group, whereas pericardial tamponade occurred in one patient in the SMC2 group (Table 2).

## Discussion

In comparison to the SMC1 the novel spiral mapping catheter SMC2 is designed as a true guidewire and is available with more electrodes and with a larger diameter. How these novel features of SMC2 impact ablation characteristics during Cryo-PVI is largely unknown. First data on SMC2 used in 40 patients undergoing Cryo-PVI was reported previously [16]. However, study population and procedural data were not compared by the authors to a control group treated with SMC1.

This is the first study to report the application of the novel spiral mapping catheter SMC2 in direct comparison to SMC1 during ablation with the second generation cryoballoon. The increased spiral tip diameter of the 25 mm SMC2 together with the two additional electrodes leads to very distinct PV electrograms, which enables higher TTI observation rates. In contrast, maneuverability is decreased in SMC2 due to a soft core wire, which has been incorporated into the novel mapping catheter. This decreased maneuverability could be overcome in many cases by stabilizing the soft shaft of SMC2 by advancing the cryoballoon catheter over the shaft of the mapping catheter thereby increasing backup. Nevertheless, it would be desirable to have a stiffer variant of SMC2 at hand, probably by incorporating a stiffer core wire into the SMC2 catheter. Despite the perceived decreased maneuverability, we did not observe an increase in procedure duration or fluoroscopy time with the 2nd generation spiral mapping catheter. However, we observed an increase in the total number of necessary cryoballoon freezes and an increase in the total freeze duration, which was mainly driven by a higher number of aborted freezes due to ineffectiveness in the SMC2 group which could be attributed to decreased stability. Taking into account that the increased TTI observation rate should have resulted in less and possibly shorter freezes, the fact that we actually observed an increase in freezes and total freeze duration might indicate, that the decreased maneuverability and stability counterbalances the advantage of higher TTI detection rates.

Real-time observation of PV isolation enables determination of the time-to-PV isolation (TTI). TTI is an independent marker of durable PV isolation or PV reconnection. Moreover, recording of the TTI enables individualization of freeze duration [10, 11, 13, 17]. We have shown previously, that TTI observation rates with the 1st generation spiral mapping catheter are higher with the 3rd generation cryoballoon as compared with the 2nd generation cryoballoon due to a shorter distal tip, which enables more proximal placement of the spiral mapping catheter within the muscular sleeve of the PV. Interestingly, with the 2nd generation spiral mapping catheter in combination with the 2nd generation cryoballoon, we find a similar rate of observed TTI as with the 1st generation spiral mapping catheter and the 3rd generation cryoballoon [9, 13].

When we compared the 20 mm 1st and 2nd generation spiral mapping catheter to the 25 mm 2nd generation spiral mapping catheter, we were able to show that it is mainly the increase in diameter and probably the addition of two electrodes, that lead to increased rates of TTI detection rates. However, when we compare the 20 mm SMC2 to the 25 mm SMC2 we do not note a difference in TTI detections rates, but this is most likely due to the small number of procedures with the 20 mm SMC2. Both better wall contact or more proximal placement because of the bigger diameter might contribute to higher TTI detection rates with the 25 mm 2nd generation spiral mapping catheter.

## Conclusions

The novel SMC2 has been designed as a true guidewire with the ability to obtain real-time PV electrograms during cryoballoon PVI. The availability of an increased diameter (25 mm) with 10 instead of 8 electrodes leads to a significant increase in the TTI observation rate. The softer shaft of SMC2 leads to decreased maneuverability and stability in patients with difficult anatomies. This leads to an increase in the necessary number of cryoballoon applications and freeze duration. Thus, even though one would expect that an increase in the TTI observation rate leads to a reduction in procedure duration and possibly fluoroscopy time when a TTI-based ablation protocol is used, neither procedure duration nor fluoroscopy time were decreased in the SMC2 group.

## Electronic supplementary material

Below is the link to the electronic supplementary material.

Angiography of the right superior pulmonary vein. Movie 1: RAO 30° (AVI 2336 kb)

Angiography of the right superior pulmonary vein. Movie 2: LAO 40° (AVI 2333 kb)

Placement of the Achieve Advance spiral mapping catheter in the RSPV Backup can be increased by advancing the cryoballoon catheter over the soft shaft of the SMC2 catheter into the PV. Movie 3: RAO 30° (AVI 18110 kb)

Placement of the Achieve Advance spiral mapping catheter in the RSPV Backup can be increased by advancing the cryoballoon catheter over the soft shaft of the SMC2 catheter into the PV. Movie 4: LAO 40° (AVI 18076 kb)
